# Dynamics of porous and amorphous magnesium borohydride to understand solid state Mg-ion-conductors

**DOI:** 10.1038/s41598-020-65857-6

**Published:** 2020-06-03

**Authors:** Michael Heere, Anna-Lena Hansen, SeyedHosein Payandeh, Neslihan Aslan, Gökhan Gizer, Magnus H. Sørby, Bjørn C. Hauback, Claudio Pistidda, Martin Dornheim, Wiebke Lohstroh

**Affiliations:** 1 Institute for Applied Materials—Energy Storage Systems (IAM-ESS), Karlsruhe Institute of Technology (KIT), 76344 Eggenstein, Germany; 2 Heinz Maier-Leibnitz Zentrum (MLZ), Technische Universität München, Lichtenbergstr. 1, 85748 Garching bei München, Germany; 3 Empa, Swiss Federal Laboratories for Materials Science and Technology, 8600 Dübendorf, Switzerland; 4 German Engineering Materials Science Centre (GEMS) at Heinz Maier-Leibnitz Zentrum (MLZ), Helmholtz-Zentrum Geesthacht GmbH, Lichtenbergstr. 1, 85748 Garching, Germany; 5 Institute of Materials Research, Materials Technology, Helmholtz-Zentrum Geesthacht, D-21502 Geesthacht, Germany; 6 Department for Neutron Materials Characterization, Institute for Energy Technology, NO-2027 Kjeller, Norway

**Keywords:** Energy, Energy science and technology

## Abstract

Rechargeable solid-state magnesium batteries are considered for high energy density storage and usage in mobile applications as well as to store energy from intermittent energy sources, triggering intense research for suitable electrode and electrolyte materials. Recently, magnesium borohydride, Mg(BH_4_)_2_, was found to be an effective precursor for solid-state Mg-ion conductors. During the mechanochemical synthesis of these Mg-ion conductors, amorphous Mg(BH_4_)_2_ is typically formed and it was postulated that this amorphous phase promotes the conductivity. Here, electrochemical impedance spectroscopy of as-received γ-Mg(BH_4_)_2_ and ball milled, amorphous Mg(BH_4_)_2_ confirmed that the conductivity of the latter is ~2 orders of magnitude higher than in as-received γ-Mg(BH_4_)_2_ at 353 K. Pair distribution function (PDF) analysis of the local structure shows striking similarities up to a length scale of 5.1 Å, suggesting similar conduction pathways in both the crystalline and amorphous sample. Up to 12.27 Å the PDF indicates that a 3D net of interpenetrating channels might still be present in the amorphous phase although less ordered compared to the as-received γ-phase. However, quasi elastic neutron scattering experiments (QENS) were used to study the rotational mobility of the [BH_4_] units, revealing a much larger fraction of activated [BH_4_] rotations in amorphous Mg(BH_4_)_2_. These findings suggest that the conduction process in amorphous Mg(BH_4_)_2_ is supported by stronger rotational mobility, which is proposed to be the so-called “paddle-wheel” mechanism.

## Introduction

Energy storage is one of the grand challenges for present and future generations. In recent years, intermittent renewable energy production has increased worldwide resulting in a high demand for energy storage systems. “Beyond Li-batteries”, which are all-solid-state batteries with alternative working ions, including Na and Mg, are considered a promising alternative as they are cheaper and with respect to their natural abundancy more sustainable. However, the transport properties of larger Na^+^ or double-charged Mg^2+^ are challenging and directly correlated to the underlying crystal structure and dynamics. Understanding the accompanying structural and dynamic changes as well as finding high-performance cathode materials remain bottlenecks for the improvement of Mg-ion batteries^1^.

Mg-ion batteries (MIBs) have several advantages compared to Li-ion technology^2^. For instance, the low electrochemical potential of −2.4 V (vs. standard hydrogen electrode (SHE)) is close to the one of Li with −3.0 V of Li/Li^+^, which allows for high cell voltages. Furthermore, Mg metal has a higher volumetric capacity of 3833 mA·cm^−3^ compared to 2036 mAh·cm^−3^ of Li metal, and magnesium has a higher natural abundancy of more than 2% in the earth crust (compared to 0.006% for lithium). Mg seems beneficial due to its non-toxicity, easy machining and handling properties^3,4^. Although a recent study suggests the appearance of Mg dendrites^5^, Mg is much less prone to form dendrites than Li^6^, where hazardous Li plating is a major safety concern. Thus, ‘pure’ Mg metal, with a very high capacity, could be used as safe and reliable anode material.

In 2017, Mohtadi and Orimo stated that the present research on complex metal hydrides is experiencing a “renaissance as energy materials”^7^. Nevertheless, for the success of future complex metal hydride-based research, the development of highly conductive electrolytes and electrodes is one of the main requirements for a successor of the Li-ion battery^8^. A first Mg-ion electrolyte based on complex metal hydrides was reported in 2012. Mohtadi *et al*. demonstrated the possibility to employ magnesium tetrahydroborate, colloquially referred to as magnesium borohydride Mg(BH_4_)_2_, dissolved in dimethoxy ethane (DME) in a rechargeable magnesium battery^9^. Shortly after, Zhao-Karger *et al*. published an ionic Mg electrolyte based on the precursor Mg(BH_4_)_2_ with the reported reaction product being Mg(BR_4_)_2_ in DME (R = –OCH(CF_3_)_2_). This electrolyte showed the highest, so far, reported electrochemical stability window of 4.3 V while being stable in air, and an ionic conductivity of 0.011 S·cm^−1^ in a 0.3 M DME solution^10^. Recently, a new compound synthesized from Mg(BH_4_)_2_ and ethylenediamine (C_2_H_8_N_2_, ‘en’) was reported to have a high magnesium ion conductivity of up to 6·10^−5^ S·cm^−1^ at 343 K in the solid state^11^, while a follow up with different organic complexes reported details on conductivities with focus on Mg(BH_4_)_2_-diglyme_0.5_^12^. The authors reported that the amorphous Mg(BH_4_)_2_ phase has a beneficial influence on the conductivities. Amorphization has also been reported to be beneficial for ionic conductivity in other material classes such as glassy solid electrolytes based on Li_3_PS_4_^13,14^. Returning to complex metal hydrides: In 2014, Mg(BH_4_)_2_ and Mg(BH_4_)(NH_4_) were proven to be “solid state” Mg ion conductors^15^ while recently, Le Ruyet *et al*. reported a correlation between an amorphous phase found in a Mg−B − N − H system by NMR with a high conductivity of 3·10^−6^ S·cm^−1^ at 373 K for a solid-state Mg conductor based on Mg(BH_4_)(NH_2_)^16^. In general, the work on Mg−B − N − H systems and the exploration of for instance dihydrogen bonds^17^ seems to increase with a recent study even showing a conductivity of σ = 3.3·10^−4^ S cm^–1^ at T = 353 K for Mg(BH_4_)_2_·NH_3_^18^.

Mg(BH_4_)_2_ seems very suitable as a precursor for Mg-ion electrolytes and hence the correlation between amorphous Mg(BH_4_)_2_ and a high ion conductivity deserves further attention. The present work addresses the structure and dynamics of porous (γ–phase) and amorphous Mg(BH_4_)_2_. Hydrogen dynamics in both materials are studied on the picosecond timescale using quasi-elastic neutron scattering. Moreover, the materials are investigated by synchrotron X-ray powder diffraction and total scattering, which were employed to elaborate the local structures despite the lack of long-range order through pair distribution function (PDF) analysis. Especially, PDF analysis is very well suited to give insights into the local structure of amorphous materials. The conductivity is determined and the results are discussed in view of the structure and dynamics in porous and amorphous Mg(BH_4_)_2._

## Results and Discussions

**Figure 1:**
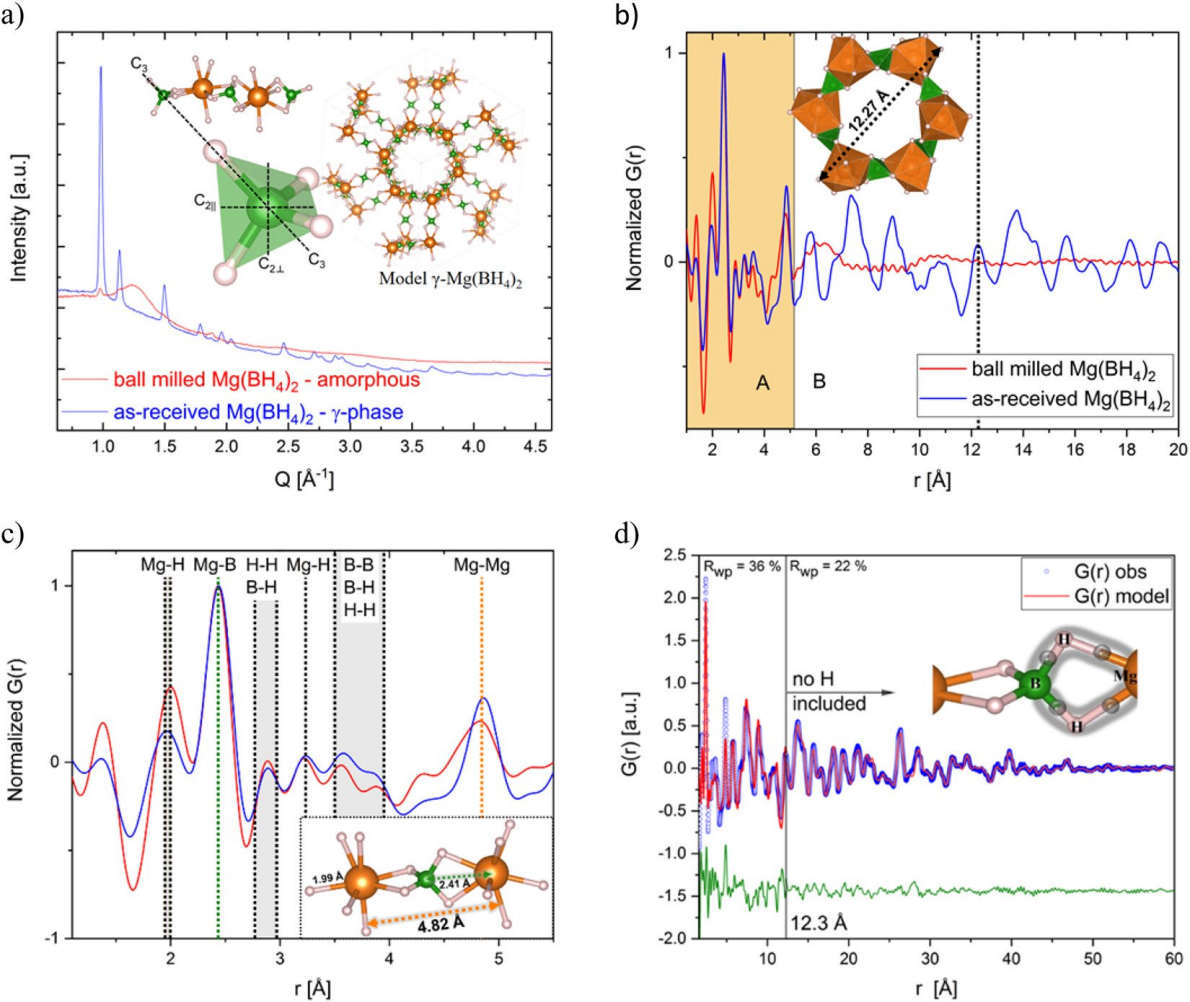
(**a**) SR-PXD of γ-Mg(BH_4_)_2_ (blue curve) and amorphous-Mg(BH_4_)_2_ (red curve). λ = 0.2072 Å. Inset in the upper left is showing three [BH_4_] tetrahedra in their respective Mg setting and a magnification into one tetrahedron and its rotational axes are shown. C_3_ is the 3-fold 120° axis and C_2∥_ and C_2⊥_ are the 2-fold 180° axis. The inset image shows the crystal structure of γ-Mg(BH_4_)_2_ with one interpenetrating channel as reported in ref. ^19^. Spheres in orange: Mg-, green: B- and grey: H-atoms. (**b**) PDF obtained from total scattering data collected at P02.1 (DESY) of amorphous and crystalline γ-Mg(BH_4_)_2_. λ = 0.20723 Å, inset: One 1D channel of the structure with 12.27 Å diameter. (**c**) Peaks of the local structure of the amorphous PDF agree well with the crystalline one up to ~5.1 Å. The last coinciding peak is the Mg – Mg distance which is marked in orange in the figure and the structural model inset. The most intense peak corresponds to the Mg-B bond (green). (**d**) Real space Rietveld fitting of the PDF of γ-Mg(BH_4_)_2_. The PDF was fitting using two different models. Details can be found in the text. Inset: Sketch of a three-centre-two-electron-bond.

The characterization of as-received and ball milled Mg(BH_4_)_2_ by synchrotron radiation powder X-ray diffraction (SR-PXD) and synchrotron X-ray total scattering experiments with corresponding PDF analysis is shown in Fig. 1. Figure 1a confirms the highly symmetric cubic structure with space group Id–3a for as-received γ-Mg(BH_4_)_2_, which we refer to as “crystalline”^19^. A single Mg atom is coordinated by the edges of four tetrahedral [BH_4_] groups. γ-Mg(BH_4_)_2_ has a 3D net of interpenetrating channels of ~12.3 Å outer diameter giving a porosity of ~33%. The ball milled Mg(BH_4_)_2_ shows diffuse scattering apart from minor Bragg peaks of γ-Mg(BH_4_)_2_, indicating a mostly X-ray amorphous phase, which we refer to as “amorphous”. Therefore notations such as “as-received” (ar) for the crystalline γ-phase as well as “ball milled” (bm) for the amorphous-Mg(BH_4_)_2_ will be used interchangeably.

The PDFs of the amorphous and crystalline γ-Mg(BH_4_)_2_ are depicted in Fig. 1b. The PDF of the amorphous sample can be divided in two main parts, A and B, with r_A_ < 5.1 Å representing the internal local structure of the main constituents or building blocks and the region 5.1 Å < r_B_ < 12.3 Å, revealing insights into the interplay (coherence) between the building blocks. Besides the first Mg – H bond, which will be discussed in more detail, the local structure of the amorphous sample agrees well with the crystalline one up to ~5.1 Å. Above ~5.1 Å, a slight oscillation is still observable, and with the aforementioned 3D net of interpenetrating channels of 12.27 Å diameter, this could indicate that the fundamental structure of the amorphous sample is still formed by these channels, even though less well-ordered. Only above ~12.3 Å the structure is completely random resulting in a featureless PDF. The good agreement of the local structure up to the first Mg – Mg distance at 4.82 Å supports spectroscopic results by Filinchuk *et al*. that the main building blocks of the structure are Mg – BH_4_ – Mg units (Fig. 1c)^19^ and that their reported X-ray amorphous phase revealed similarities to the local structure of the γ- and δ-Mg(BH_4_)_2_^19^. The B-B distances are less pronounced in the PDF of the amorphous samples, indicating a higher amount of disorder and less correlation between the tetrahedra. It has to be noted, that even hydrogen bonds can be observed in the PDF, due to the fact that H has a formal oxidation state of –1 and therefore a notable electron density. However, to understand the different intensities of the Mg – H peak of the amorphous and the crystalline sample, two peculiarities have to be kept in mind. First, just as in B_2_H_6_, Mg – BH_4_ – Mg are interconnected via three-centre-two-electron bonds (tc-te). This means that the two electrons are “smeared out” over three atoms as depicted in the inset of Fig. 1d. Second, only information about electron densities are accessible using X-ray scattering methods. Consequently, in case of an X-ray PDF, the peak of corresponding tc-te bonds is expected to have only half of the intensity and being broadened, due to the delocalization of electrons. The fact that the Mg – H peak of the amorphous sample is more intense and narrower, reveals the presence of fewer tc-te bonds and therefore, more terminating Mg – H bonds.

To account for the bonding situation, the occupancy of H atoms was reduced to 0.5 in the models to fit the PDF. To refine the structural model of γ-Mg(BH_4_)_2_ the so called real space Rietveld approach was used^20^. It was not possible to fit the PDF over the whole range, therefore, it was divided into a short range ordering (SRO) and a long range ordering (LRO) part, being r < 12.3 Å ( = diameter of a interpenetrating channel) and r > 12.3 Å, respectively. No difference was observed if H was included in the model to fit the LRO. The modelled PDF agrees reasonably well up to 60 Å, but fitting the SRO certainly needs more attention.

Various models were used to describe the SRO features in the PDF (Fig. A1, supplementary information SI). All the models are based on the cubic γ-Mg(BH_4_)_2_ structure. All peak positions agree well, as already indicated in Fig. 1d. Nevertheless, especially the Mg – Mg peaks (4.82 Å, 7.40 Å, 8.85 Å) differ in their relative intensities. More precisely, either the intensity of the higher orders or the intensity of the first peaks can be fitted by using large or small anisotropic temperature factors, model 1 or 2, respectively. This reveals, that the Mg – Mg correlation within the interpenetrating channels cannot be described in accordance with the cubic symmetry. We therefore, carefully allowed the Mg ions to move individually into the direction the temperature factors were pointing, *e*.*g*. into the channels (model 3), leaving their positions given by the cubic symmetry. To simplify this model, the H atoms were not included and isotropic temperature factors were utilized. Comparing all three models in Fig. A1, model 3 seems the best to describe the local Mg disorder for the SRO. In future, neutron powder diffraction measurements are planned especially within the frame of the “Energy research with Neutrons (ErwiN)” instrument at the MLZ, Germany^21^.

*In situ* SR-PXD and thermogravimetric and differential thermal analysis (TG-DTA) are shown in Fig. A2 and A3 in the SI. TG-DTA data are presented in Fig. A3, showing the various phase evolutions in the investigated samples until 500 K, which all correspond to the literature^22^. The γ-Mg(BH_4_)_2_ transforms into ε-Mg(BH_4_)_2_ at T_peak_ = 439 K and into β’-Mg(BH_4_)_2_ at T_peak_ = 470 K. Both are endothermic events (blue curve in Fig. A3). The ball milled amorphous-Mg(BH_4_)_2_ transforms via an exothermic event first into γ-Mg(BH_4_)_2_ at 372 K (crystallization), followed by the endothermic transitions towards the ε- and β′-phase. The latter events are slightly reduced in peak temperature to 432 K and 461 K compared to the as-received sample, at the same heating rate, which is in good agreement with literature^22^. The decomposition reactions are not reported here but generally start from ~470 K with the release of hydrogen and the formation of non-crystalline, intermediate Mg–B–H species of different stoichiometry^23,24^.

**Figure 2:**
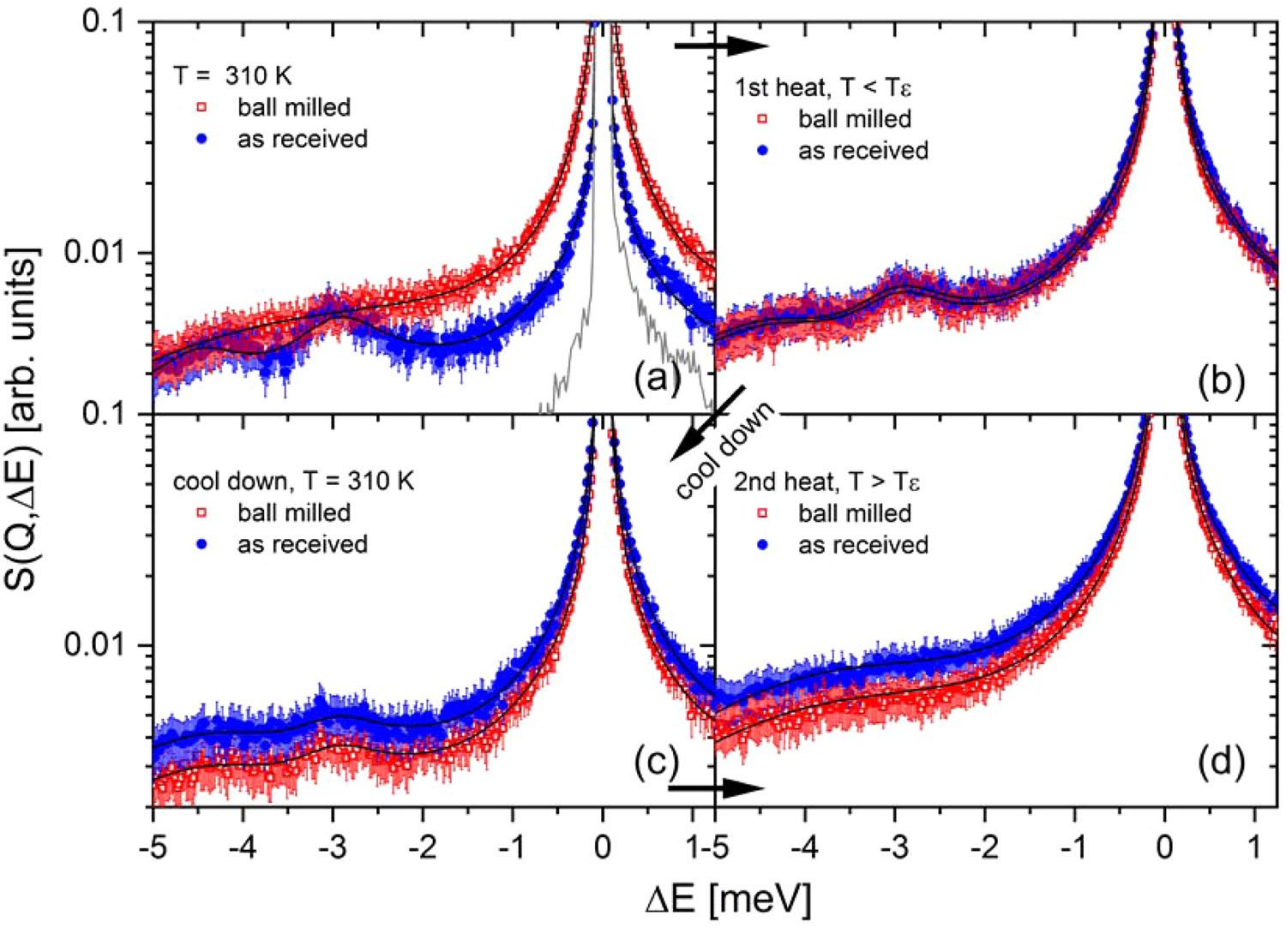
Mg(BH_4_)_2_ in crystalline γ-modification (as-received (ar), blue circles), and amorphous modification (ball milled (bm), red squares). S(Q, ∆E) measured at λ_1_ = 5 Å, Q = 1.35 Å^−1^. (**a**) T = 310 K, the solid grey curve shows the measured resolution (res) function at 3.5 K. (**b**) T(ar) = 419 K, T(bm) = 410 K, (**c**) 310 K, (**d**) T(ar) = 435 K, T(bm) = 431 K. The solid black curves represent the fit to the data.

**Figure 3:**
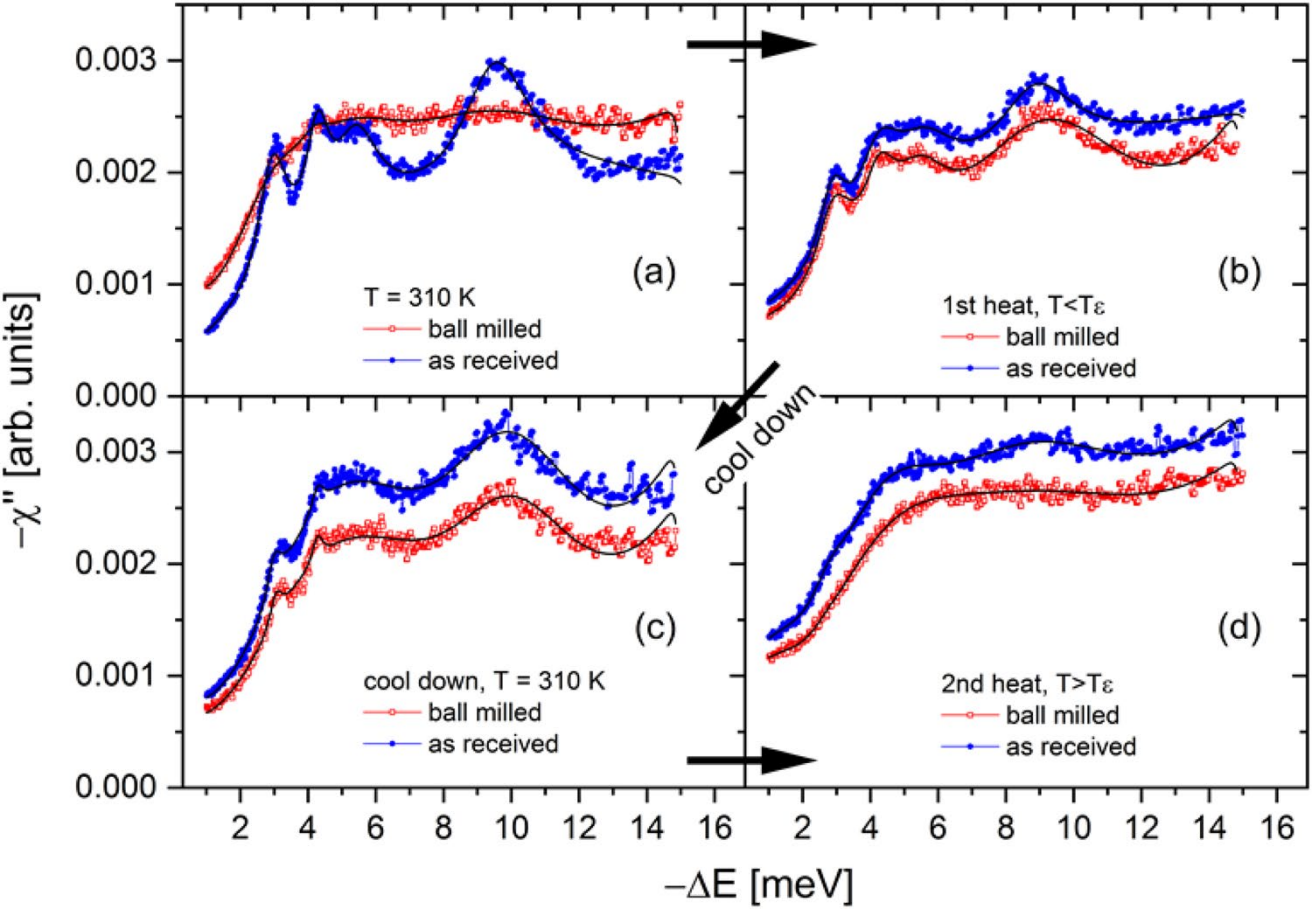
Dissipative part of the dynamical susceptibility (−χ”) as a function of energy transfer –∆E [meV] and temperatures of (**a**) 310 K, (**b**) the temperature was below the ε-phase transition, T < T_ε_ in detail T(ar) = 419 K, T(bm) = 410 K. (**c**) Followed by a cooling to T=310 K and (**d**) a second heating procedure above the ε-phase transition temperature, T > T_ε_ in detail T(ar) = 435 K, T(bm) = 431 K. The solid black curves represent the fit to the data. Blue circles: as-received γ-Mg(BH_4_)_2_. Red squares: ball milled Mg(BH_4_)_2_.

**Figure 4:**
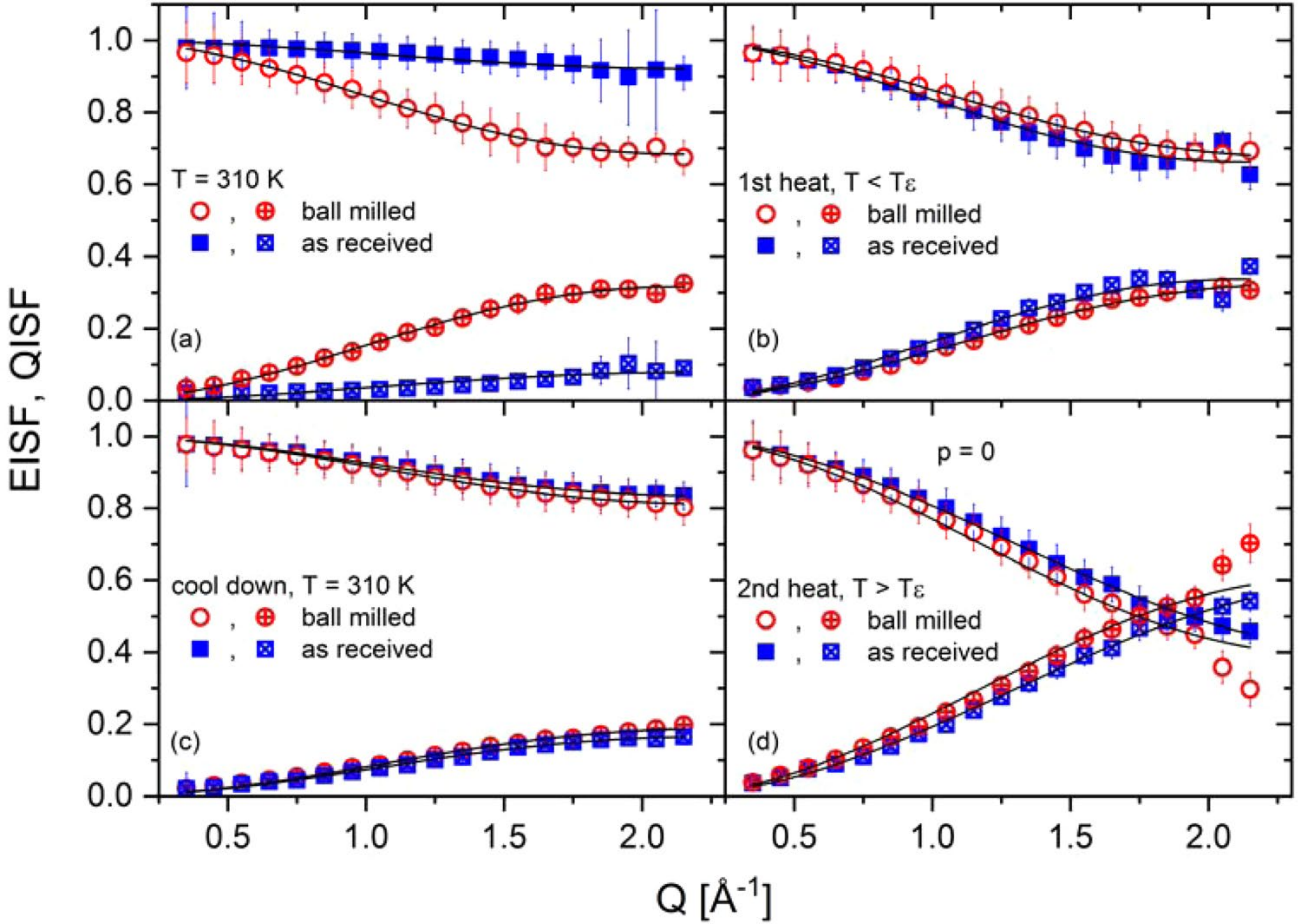
Elastic incoherent scattering function (EISF) and quasi-elastic incoherent scattering function (QISF) as a function of four different temperatures for as-received γ-Mg(BH_4_)_2_ and ball milled Mg(BH_4_)_2_. (**a**) 310 K, (**b**) the temperature was below the ε-phase transition, T < T_ε_ in detail T(ar) = 419 K, T(bm) = 410 K. (**c**) Followed by a cooling to T=310 K and (**d**) a second heating procedure above the ε-phase transition temperature, T > T_ε_ in detail T(ar) = 435 K, T(bm) = 431 K. Blue circles: as-received γ-Mg(BH_4_)_2_. Red squares: ball milled Mg(BH_4_)_2_. EISF top curves, QISF bottom curves. λ = 5 Å.

In order to investigate the internal dynamics of γ- and amorphous Mg(BH_4_)_2_, quasi-elastic neutron scattering (QENS) experiments have been conducted. QENS is a powerful technique to investigate stochastic motions such as diffusion or jump rotations in condensed matter, which result in characteristic broadening of the elastic line at zero energy transfer (∆E = 0 meV). In general, a good overview of dynamic investigation of complex metal hydrides can be found in ref. ^25^. Figure 2 shows the obtained scattering function S(Q, ∆E) at λ_1_ = 5 Å for as-received and ball milled Mg(BH_4_)_2_ at various temperatures. The results at 310 K (Fig. 2a) indicate that the low energy spectra – both the quasi-elastic and the inelastic contribution – are strongly dependent on the local structure as can be seen by the comparison of as-received γ-Mg(BH_4_)_2_ and ball milled amorphous material. While as-received Mg(BH_4_)_2_ at 310 K shows almost no quasi-elastic scattering around the elastic peak, the ball milled material exhibits significant broadening of the elastic peak indicating a higher rotational mobility of the [BH_4_] units (for details see Figs. 3 and 4). Furthermore, distinct low energy (vibrational) inelastic peaks are observed in the as-received γ-species, which cannot be resolved in the ball milled sample. The energy of the inelastic excitations is constant over all probed Q-values.

Both samples underwent two heating cycles. The first heating went up to temperatures below the ε-phase transition (419 K for ar and 410 K for bm), the second one above the phase transformation temperature (435 K for ar and 431 K for bm). After the first heating a crystallization reaction towards γ-phase occurred in the ball milled sample, which resulted in a very similar scattering function compared to the as-received sample (Fig. 2b). The subsequent cooling step to 310 K was essential to compare the two samples near room temperature (after crystallization of bm phase to the γ-phase). The quasi-elastic signal and inelastic scattering in Fig. 2c, measured at 310 K, seems to be an average of those depicted in Fig. 2a. The strong quasi-elastic signal of the ball milled sample decreased while the as-received quasi-elastic signal increased. Simultaneously, the inelastic scattering increased for the ball milled sample and decreased for the as-received sample.

Figure 3 presents the imaginary (or dissipative) part of the dynamical susceptibility –χ” as a function of energy transfer −∆E [meV] at the same experimental conditions as reported in Fig. 2. The dissipative part of the dynamical susceptibility is connected to the scattering function S(Q, ΔE) through the dissipation - fluctuation theorem by Eq. 1:

1 $$\frac{1}{\pi }\chi {\prime\prime} =\frac{S(Q,-\Delta E)}{{n}_{B}}$$

where *n*_*B*_ is the Bose occupation factor, ${n}_{B}={\left[\exp \left(\frac{\Delta E}{kT}\right)-1\right]}^{-1}$, and *k* is the Boltzmann constant^26^. A plot of −χ” emphasizes weak features in the inelastic regime, and the analysis of the position and width of the vibrational peaks was used as input for the in depth - analysis of the quasielastic contribution. As there is no Q-dependence in the inelastic spectra, the data have been summed over all Q values to increase statistics. In Fig. 3a as-received γ-Mg(BH_4_)_2_ shows distinct contributions at –∆E ~ 3, 4.2, 5.4 and 9.4 meV. In contrast, –χ” of ball milled Mg(BH_4_)_2_ has less pronounced features and exhibits an increased intensity at low energy transfer due to the strong quasi-elastic contribution. In Fig. 3b, after first heating, the contributions in both phases become quite similar, suggesting the transformation of the amorphous phase into the γ-phase, which is in good agreement with data presented before (DTA, SR-PXD). In Fig. 3c,d, showing the imaginary part of the dynamical susceptibility –χ” after the first heating and during the second heating and that there are no noticeable differences observed between the two samples. Although the nature of distinct contributions (or modes) in Fig. 3a (ar) has not been identified yet (and it is not subject of this study), the features are resembling rigid unit motions as observed in various modifications of SiO_2_, which results from small scale rotations of interconnected SiO_4_ tetrahedra^27^. Note that the librational frequency of the BH_4_ tetrahedra is at much higher energies around 65 meV^28,29^. The existence of rigid unit motions suggests a coupling of adjacent BH_4_ tetrahedra via a connecting Mg ion, as there is no direct BH_4_ – BH_4_ bond in the structure (Fig. 1).

In Fig. A8a, the dissipative part of the dynamical susceptibility of as-received γ-Mg(BH_4_)_2_ at 310 K is compared to the one of α-Mg(BH_4_)_2_ at 300 K^30^, illustrating that the external modes in γ-Mg(BH_4_)_2_ are even softer, but clearly as rich or even richer in features as α-Mg(BH_4_)_2_. The plot highlights the dependency of the excitations on the long range structure of the respective Mg(BH_4_)_2_ polymorph. (Note, that the two spectra have been measured at different instrument settings, thus, the decreased half-width at half maximum (HWHM) of γ-Mg(BH_4_)_2_ compared to the α-Mg(BH_4_)_2_ is partially due to the better energy resolution in the present work). After heating to 460 K, the spectra depicted in Fig. A8b resembles very much the one obtained for β-Mg(BH_4_)_2_ from ref. ^31^, meaning that the β-phase transition is completed as shown in SI Fig. A4.

The black curves in Fig. 3 are the fitting of $-\chi \text{'}\text{'}$ and these values were employed to determine the characteristic frequencies of the underlying inelastic features. The results are given in Fig. A7 (SI). Remarkably, the values of the characteristic energies *E*_*D,i*_ for both samples are fairly constant over the entire temperature range. During the first heating, at 310 K, the amorphous sample exhibits a larger damping *γ*_*D,i*_ as compared to the as-received one, while no such differences are observed for the second heating procedure.

Using *E*_*D,i*_ as fixed parameters, *S(Q*, Δ*E)* for both samples have been analyzed according to Eq. 2.

2 $$\begin{array}{rcl}{S}_{meas}(Q,\Delta E) & = & Res\otimes {S}_{rot}\otimes {S}_{vib}\\  & = & Res\otimes [D(Q){A}_{0}(Q)\delta (\Delta E)+D(Q){A}_{1}(Q){L}_{1}({\Gamma }_{1},\Delta E)+{S}_{inel}]\end{array}$$

Two Lorentzian functions, L_1_ and L_2_ with HWHM Γ_1_ and Γ_2_, and the elastic line are needed in addition to the damped harmonic oscillators, DHO (with E_D__,__i_ (i=1-4)) to describe the data satisfactorily. The results of the fits are shown in Fig. 2 as solid black curves. For both Lorentzians, the HWHM is independent of Q, which indicates localized motions. For the analysis, L_1_ (smaller HWHM) was ascribed to the quasi-elastic contribution originating from rotational motions of the BH_4_ units while L_2_ was considered part of the inelastic scattering contribution originating from an (additional) overdamped harmonic oscillator. Since the intensity of the second Lorentzian is much weaker compared to L_1_ its origin cannot be a faster rotation around a different axis, in this case, one would expect the contribution to be more intense than L_1_. There is only one crystallographic site of the BH_4_ units in γ-Mg(BH_4_)_2_. Nevertheless, characteristic times τ_1,2_ = ħ/Γ_1,2_ were obtained from the fit of the two Lorentzian functions as a function of temperature which are shown in Fig. A9. While Fig. A9a shows the results of the first heating cycle for both as-received γ-Mg(BH_4_)_2_ and ball milled amorphous-Mg(BH_4_)_2_, Fig. A9b depicts the results of the second heating cycle. At 310 K, τ_1_ and τ_2_ are lower in γ-Mg(BH_4_)_2_ compared to amorphous-Mg(BH_4_)_2_. During the first heat, the amorphous sample first shows a decrease of τ_1_ and τ_2_ when heating from 310 K to 355 K, indicating that jump rates become higher. Upon further heating and the onset of crystallization, both τ_1_ and τ_2_ steadily increase and at 420 K both samples show comparable (slower) jump rates. During the second heating, no such differences are observed and both samples follow the same trend: Up to 420 K, τ_1_ and τ_2_ are almost constant and only towards the ε-phase transition at 423 K, a continuous decrease of τ_1,2_ is observed. The solid line in Fig. A9b illustrates an Arrhenius type fit to τ_1_ (ascribed to rotational motions), i.e. τ = τ_0_ exp(E_a_/RT), which gives an apparent activation energy in the ε- and β’-phase of *E*_*a*_ = 3.2 ± 0.5 kJ/mol (33.2 ± 5.2 meV) and τ_0_ = 1.4 ps. In contrast, in γ-Mg(BH_4_)_2_ τ_1_ remained almost constant with increasing temperature, and no activation energy could be determined. This observation is in agreement with NMR data on the γ-phase^32^, which showed that the apparent activation energies for rotation are much higher in the γ-phase compared to the β-modification.

In general, the temperature dependence of the jump frequency τ_1_ was used to calculate the activation energy E_a_ in reported literature as well. Blanchard *et al*. described three thermally activated processes in the β-Mg(BH_4_)_2_ polymorph^33^. The first one with an activation energy of E_a_ = 39 ± 0.5 meV was confirmed by Silvi *et al*. who reported E_a_ = 36 ± 3 meV^31^. This mode had been assigned to the rotation around the C_2||_ axis, as it is the energetically more favourable rotation of the [BH_4_] bidentate configuration. Furthermore, in ref. ^33^, two modes have been reported at E_a_ = 76 ± 5 meV and E_a_ = 214 ± 4 meV for β-Mg(BH_4_)_2_ with an assignment to the C_2||_ and C_3_ rotation axis. In ref. ^29^ the energy of rotation have been reported to peak around 65 meV and have been assigned to rigid liberations of the [BH_4_]^–^ tetrahedra after ref. ^28^ and been confirmed as well in ref. ^34^ with the same trend observed.

Interestingly, our relaxation times of the amorphous-Mg(BH_4_)_2_ can be compared to those of the solid state Mg conductor Mg(BH_4_)_2_-diglyme_0.5_^12^, where it was reported that an amorphous component was present. The authors hypothesized that it was amorphous-Mg(BH_4_)_2_. It can be observed that the decrease in relaxation times reported here for both τ_1_ and τ_2_ up to 360 K can be found in that particular study as well. This means that there is evidence of an amorphous Mg(BH_4_)_2_ in their sample, although no attempt on heating towards crystallization was reported. If that would have happened, it would most likely have shown a decrease in the temperature dependent characteristic relaxation times.

From the fit of *S(Q*, Δ*E)*, the intensities I_el_, and I_qe_ of the elastic (el) and quasi-elastic (qe) contribution to the scattering have been obtained. From these, the Elastic (A_0_) and Quasi-elastic (A_1_) Incoherent Structure Factors can be calculated (with A_0_ = 1 – A_1_) according to Eq. 3:

3 $$EISF={A}_{0}=\frac{{I}_{el}}{{I}_{el}+{I}_{qe}}=\frac{D(Q){A}_{0}(Q)}{D(Q){A}_{0}(Q)+D(Q){A}_{1}(Q)}=1-QISF=1-{A}_{1}$$

The results are shown in Fig. 4. As aforementioned, there is a marked difference between amorphous Mg(BH_4_)_2_ and γ-Mg(BH_4_)_2_ at 310 K. The latter shows almost no quasi-elastic intensity, and accordingly, the EISF is close to one. Once the amorphous sample is crystallized, the observed structure factors are very similar for both samples. The EISF and QISF have been analyzed assuming hindered rotations around the C_2_ or C_3_ axis of the BH_4_ tetrahedra, which can be modelled as described in Eq. 4:

4 $$EISF=p+(1-p)\frac{1}{2}\,\left[1+{j}_{0}\left(Q\frac{2\sqrt{2}}{\sqrt{3}}{d}_{B-H}\right)\right]$$

The solid lines in Fig. 4 are fits according to Eq. 4 with the free parameter p, the fraction of hindered rotations, and d_B–H_, the boron–hydrogen distance. Values obtained for d_B–H_ vary between 1.10–1.28 Å, thus, well within the expected range for the BH_4_ tetrahedra^19^ (Note, that the sensitivity of the fit on d_B–H_ is limited). For crystalline Mg(BH_4_)_2_, the fraction of hindered rotations p exhibit a continuous increase of activated BH_4_ rotations until all C_2_/C_3_ modes are active (i.e. p = 0) at a temperature that coincides with the ε-phase transition temperature. In contrast, amorphous Mg(BH_4_)_2_ initially exhibits a larger rotational activity at 310 K, however the number of activated rotations decreases upon heating towards crystallization (see Fig. A10). One might speculate that the larger number of terminating Mg-H bonds in amorphous Mg(BH_4_)_2_ (effectively interrupting the interpenetrating channels and their ..-Mg-H_2_BH_2_-Mg-.. chains) favors stochastic rotations of the [BH_4_] units. After the sample has crystallized into the γ-phase, the dynamic properties are almost identical for both samples. The mean square displacement (<u^2^>) of the hydrogen atoms has also been obtained from the analysis: γ-Mg(BH_4_)_2_ shows an continuous increase in <u^2^> with temperature while <u^2^> of the ball milled phase (at 310 K)) is larger, and remains almost constant upon heating to 380 K (crystallization). Both samples appear to be quite similar in the temperature range 380–455 K (Fig. A11).

**Figure 5:**
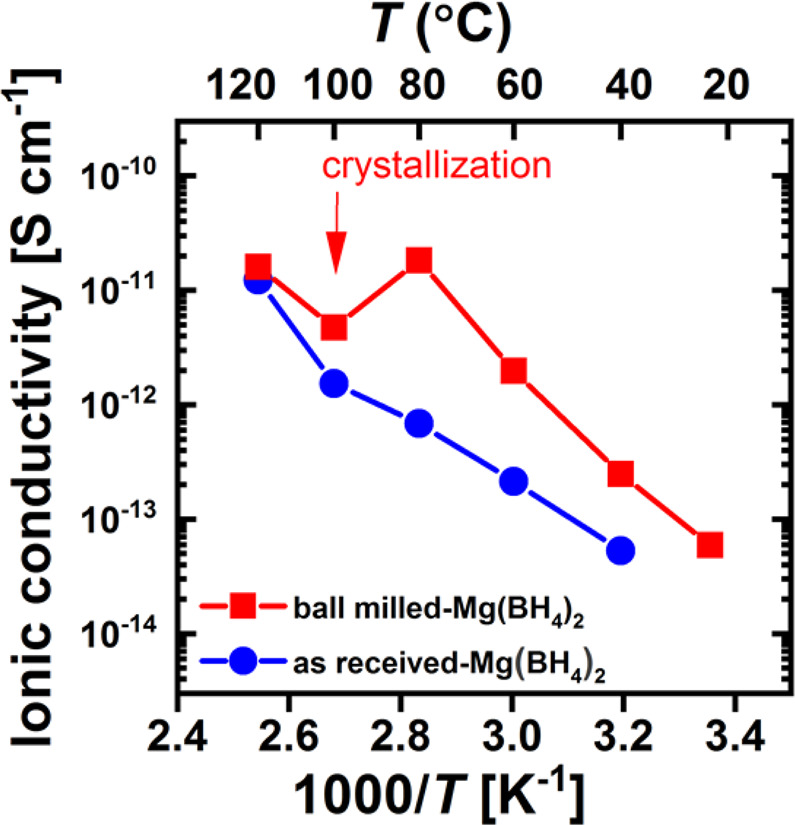
Arrhenius plot of the magnesium ion conductivity of as-received γ-Mg(BH_4_)_2_ and ball milled-Mg(BH_4_)_2_.

The temperature dependence of the magnesium ion conductivity for γ-Mg(BH_4_)_2_ and ball milled-Mg(BH_4_)_2_ was determined by electrochemical impedance spectroscopy (EIS) and is presented in Fig. 5. γ-Mg(BH_4_)_2_ shows the conductivity of 5.3·10^−14^ S·cm^−1^ at 313 K which increases to 6.9·10^−13^ S·cm^−1^ and 1.2·10^−11^ S·cm^−1^ at 353 K and 393 K, respectively, with the activation energy of 0.68 eV. Ball milled-Mg(BH_4_)_2_ shows a higher conductivity of 2.5·10^−13^ S·cm^−1^ already at 313 K and 1.8·10^−11^ S·cm^−1^ at 353 K with an activation energy of 0.95 eV. The conductivity values of the ball milled sample are higher in the entire temperature range. At 373 K, a drop in the conductivity is observed which corresponds to the aforementioned crystallization to γ-Mg(BH_4_)_2_ also shown in PXD data depicted in Fig. A12. However, our measured conductivity is low compared to earlier work were a conductivity of <10^−12^ S·cm^−1^ at 303 K was reported for pristine Mg(BH_4_)_2_^11^.

The exploration of dynamics of the host lattice goes back to the 1970s, where it was suggested that the ionic conductivity, which is a thermally activated process^35^, is proportional to the phonon spectrum^36^. The latter is caused by lattice vibrations which correlate to the elastic stiffness of the lattice. In Kraft *et al*. the authors suggest two contradicting processes for an increase in conductivity in lithium argyrodites based materials^37^. The first is the lowering in activation energy caused by a softer lattice while simultaneously a softer lattice decreases the attempt frequency of the ion jump as well as the migration entropy^37^. A softer lattice is thus not necessarily better for a solid ionic conductor^37^. In our work, we show that the number of rotating [BH_4_] units in Mg(BH_4_)_2_ increases by mechanochemical treatment and that ionic conductivity is almost 2 orders of magnitudes higher at 353 K.

The increase of the number of rotating [BH_4_] units is, what we believe, direct evidence of the so-called “paddle-wheel” mechanism^38^, which most recently has also been shown by QENS measurements in *carba*-borohydrides^39,40^. This “paddle-wheel” mechanism has been suggested by computational results for the complex [BH_4_] anion^41^. Therefore, it was suggested to be aiding the conduction process in complex metal hydrides^42–44^. Experimentally, solid-state Nuclear Magnetic Resonance and QENS measurements^45,46^ indicated that the high rotational mobility promotes super-conductivity in these materials^47,48^. Thus, the observed correlation between rotational mobility and conductivity lead to effects which are possibly flattening the energy landscape by dynamic frustration^49^.

The activation energy increased for the ball milled sample, which is contra intuitive, leaving us puzzled what the dominating process of the ion conduction process is. A decrease of the attempt frequency of the ion jump and the migration entropy will be investigated further in future. One might speculate, that the former is correlated to the local [BH_4_] rotations. During heating, the number of activated rotations in amorphous Mg(BH_4_)_2_ slowly decreases already at temperatures well below the crystallization temperature, thus potentially counteracting the thermal activation of the ion jumps (compare Fig. A10). As a remark, in our work we do change the local atomic structure possibly by misplacing Mg ions slightly (as shown in comparison of model 3 in Fig. A1 and Fig. 1d), therefore it is hard to judge if the increased conductivity stems from the change in structure or from the softer lattice. Probably a combination of both, while we are not changing any anions, therefore not changing the charge carriers.

## Conclusions

Quasi-elastic neutron scattering (QENS) studies were employed to investigate the dynamics of porous (γ-) and amorphous Mg(BH_4_)_2_. The corresponding PDFs show that the local structure of the amorphous sample agrees reasonable well with the crystalline one up to ~5.1 Å, meaning that main building blocks of the structure remain Mg – BH_4_ – Mg units. Above 5.1 Å, a slight oscillation is still observable up to ~12.3 Å, which is in good agreement with the diameter of a one dimensional channel and thus indicates that the fundamental structure of the amorphous sample is still formed by these channels, even though less well-ordered. QENS studies found a correlation in the relaxation times of the ball milled (amorphous) Mg(BH_4_)_2_ and Mg(BH_4_)_2_-diglyme_0.5_. Additionally, for Mg(BH_4_)_2_-diglyme_0.5_, a step-like increase of the mean square displacement has been reported. In contrast, the mean square displacement in our study was found to be completely linear over a broad temperature spectrum^12^.

The mechanochemical synthesis method of the recently reported solid-state Mg-ion conductors, Mg(en)(BH_4_)_2_ and Mg(BH_4_)_2_-diglyme_0.5_, tends to form amorphous Mg(BH_4_)_2_ as a byproduct^11,12^. Its influence on the conduction properties is unknown, but it was postulated that the amorphous phase is helping to increase the conductivity. Electrochemical impedance spectroscopy for ionic conductivities measurements were employed here and it was found that the conductivity of the amorphous phase is indeed ~2 orders of magnitude higher than the as-received γ-Mg(BH_4_)_2_ at 353 K. PDF analysis found similar local building blocks, thus suggesting also similar conduction pathways. QENS data showed a higher fraction of activated rotations in the amorphous sample. Thus it is postulated that the conduction process in amorphous Mg(BH_4_)_2_ is supported by rotating [BH_4_] units. Upon crystallization at 373 K, the number of rotations decreases as well as conductivity values.

This study confirms that the amorphous phase of Mg(BH_4_)_2_ has an important contribution in future Mg-ion conductors and therefore its presence needs to be taken into account for follow-up investigations.

## Experimental Methods

### Sample preparation

Mg(BH_4_)_2_ powder was purchased from Sigma–Aldrich in γ–modification (>95%) (space group *Id*–3*a*^19^) and was used as-received (ar). Amorphization of the Mg(BH_4_)_2_ was achieved by ball milled (bm) in a P6 planetary ball mill for a total of 1 h, divided in 4 times 15 min and 5 min breaks in between to avoid overheating^50^. Stainless steel vials with stainless steel–balls were used with a ball-to-powder ratio 40:1. All sample manipulations were performed under Argon atmosphere in an MBraun Unilab glove box (O_2_/H_2_O sensors were kept under 1ppm).

### Quasi-elastic neutron scattering (QENS)

were conducted at the time-of-flight spectrometer TOFTOF operated by the Technische Universität München at the Heinz Maier-Leibnitz Zentrum (MLZ) in Garching, Germany. TOFTOF has a direct geometry and employs cold neutrons^51^. The raw neutron data were normalized to the incoming flux and vanadium, corrected for background and self-shielding absorption effects. The time-of-flight data were transformed to energy transfer, and the momentum transfer Q was calculated. The obtained dynamic structure factor S(Q, ΔE) was binned into a regular grid in energy transfer (ΔE) and momentum transfer (ΔQ). Measurements were taken at λ = 5 Å incident wavelength, which gave an instrument resolution at the elastic line of 0.065 meV (FWHM = full width half maximum) and an accessible momentum transfer range of Q = 0.3–2.2 Å^−1^. All measurements were performed in transmission mode with the sample containers oriented at an angle of 135° with respect to the incoming beam. Measurements were performed in a cryofurnace at discrete temperatures of 3.5 K (resolution), and between 310 K and 460 K.

~100 mg of material was employed for each measurement with an estimated neutron transmission of ~85% (using samples with natural boron). A broad range of temperatures was chosen with two motivations: First to observe the exothermic phase transition from amorphous to the crystalline γ-modification, secondly to observe the endothermic phase transitions during heating above 423 K (ε- and β′-Mg(BH_4_)_2_ structures^24,52^). Each temperature was measured for 3–5 h, with additional 30 minutes of equilibration time.

### Thermogravimetric and differential thermal analysis (TG-DTA)

experiments were conducted using a Netzsch STA 409 C/CD analyzer. The experiments were conducted from room temperature (RT) to 563 K at 5 K min^–1^. All samples were measured within Al_2_O_3_ crucibles. The Ar flow (protective and purge gas) was 20 and 50 ml min^–1^, respectively.

### Synchrotron radiation powder X-ray diffraction (SR-PXD)

data were collected at DESY at beamline P02.1^53^. For *in situ* SR-PXD experiments the samples were contained in 0.8 mm sealed sapphire capillaries. The sample-to-detector distances and the wavelength were calibrated from a NIST silicon standard. Data were collected using a Perkin Elmer XRD1621 area detector. The exposure time was set to 10 s giving a temperature resolution of 0.83 K per pattern. The data were integrated to 1D diffraction patterns in DAWN 2^54^.

### Synchrotron X-ray total scattering

experiments were executed at room temperature. Data were collected at the high energy powder diffraction and total scattering beamline P02.1 with photon energies of 60 keV (λ = 0.20723 Å)^53^. Sample-detector-distance was 220 mm. The scattering data was acquired using a Perkin Elmer XRD1621 (200 × 200 µm^2^ pixels) area detector. The total exposure time was 30 minutes. The integration of 2D pattern was performed using DAWNscience^54^. An empty 1 mm glass (Hilgenberg Glass no. 10) capillary was measured under the same conditions and subtracted from the measured data. To account for instrumental contribution silicon standard (NIST 640a) was measured. The corresponding pair distribution function was calculated using the program PDFgetX3 with a Q_max_ = 23.8 Å^−1^^55^. In future, neutron powder diffraction measurements are planned especially within the frame of the “Energy research with Neutrons (ErwiN)” instrument at the MLZ, Germany^21^.

### Attenuated total reflection IR (ATR-IR)

measurements were performed using a -Agilent Technologies Cary 630 infrared spectrometer with a diamond crystal inside an Ar-filled glove box. The spectra were obtained in the wavenumber range of 4000-650 cm^–1^ with a resolution of 4 cm^−1^ at RT. 300 scans were averaged for each spectrum and the background. IR spectra were ATR corrected using commercial spectroscopic software OPUS. The samples were measured without any dilution.

### Mg ionic conductivity measurements

were measured by electrochemical impedance spectroscopy (EIS) using a Novocontrol potentiostat with a voltage amplitude of 50 mV in the frequency range from 1 MHz to 0.01 Hz. Powder samples (150, 112 mg) were compressed in pellets with thicknesses of 1.21 and 1.34 mm and diameters of 15.5 and 12.5 mm using an axial hydraulic press with a pressure of 282 and 80 MPa  for γ-Mg(BH_4_)_2_ and ball milled Mg(BH_4_)_2_ respectively. The pellets were sandwiched between indium foils to improve the contact between the sample and the brass electrodes and were mounted in an airtight cell (BDS1308). The samples were measured from 20 to 120 °C. A R1/Q1 model was used to fit the conductivity data (Fig. A13). The relative densities of the pellets are presented in Tab. S1.

## Supplementary information

Supporting Information.
