# Supplementary material for: Dynamics of porous and amorphous magnesium borohydride to understand solid state Mg-ion-conductors
Source: Sci Rep. 2020 Jun 3;10:9080. doi: 10.1038/s41598-020-65857-6 (PMC7270138; doi:10.1038/s41598-020-65857-6)
Supplement: Supplementary file 1 — Supporting Information. [file 41598_2020_65857_MOESM1_ESM.pdf]

Supporting Information

to the manuscript

Dynamics of porous and amorphous magnesium borohydride to understand solid state Mg-ion-conductors

Michael Heere[1,2*], Anna-Lena Hansen[1], SeyedHosein Payandeh[3], Neslihan Aslan[4], Gökhan Gizer[5], Magnus H. Sørby[6], Bjørn C. Hauback[6], Claudio Pistidda[5], Martin Dornheim[5], and Wiebke Lohstroh[2*]

[1] Institute for Applied Materials—Energy Storage Systems (IAM-ESS), Karlsruhe Institute of Technology (KIT), 76344 Eggenstein, Germany

[2] Heinz Maier-Leibnitz Zentrum (MLZ), Technische Universität München, Lichtenbergstr. 1, 85748 Garching b. München, Germany.

[3] Empa, Swiss Federal Laboratories for Materials Science and Technology, 8600 Dübendorf, Switzerland.

[4] German Engineering Materials Science Centre (GEMS) at Heinz Maier-Leibnitz Zentrum (MLZ), Helmholtz-Zentrum Geesthacht GmbH, Lichtenbergstr. 1, 85748 Garching, Germany

[5] Institute of Materials Research, Materials Technology, Helmholtz-Zentrum Geesthacht, D-21502 Geesthacht, Germany

[6] Department for Neutron Materials Characterization, Institute for Energy Technology, NO-2027 Kjeller, Norway

Correspondence and requests for materials should be addressed to M.H. and W.L (email: Michael.Heere@kit.edu; Wiebke.Lohstroh@frm2.tum.de)

Keywords: magnesium borohydride, quasi-elastic neutron spectroscopy, solid state Mg-electrolyte, solid-state conductors, total scattering, pair distribution function

Neutron data analysis

Due to the high incoherent scattering cross section of hydrogen, the signal registered at the detector is predominantly from hydrogen. Generally, in the incoherent approximation, the scattering function S(Q,ΔE) for particles performing simultaneous but independent motions can be described as:

$$S(Q,\Delta E) = S_{trans}(Q,\Delta E) \otimes S_{rot}(Q,\Delta E) \otimes S_{vib}(Q,\Delta E)$$

i.e. it is given by the convolution of the scattering functions for translational diffusion, rotational and vibrational motions, respectively. In borohydrides, in the temperature range studied here, relevant for the analysis are jump rotational diffusion processes of the BH_4 tetrahedra and fast vibrational processes, but no translational diffusion processes have been observed below 500 K, similarly to α- and β-$Mg(BH_4)_2$ [1,2] and $LiBH_4$ [3].

For the BH_4 tetrahedra, rotations around the C_2 or C_3 symmetry axis are most likely, and the scattering function for this rotation can be described by:

$$S(Q,\Delta E) = A_0(Q)\delta(\Delta E) + A_1 L_1(\Gamma_1)$$

i.e. the sum of an elastic contribution and a Lorentzian $L_1(\Gamma_1)$ that is characterized by its half-width at half maximum (HWHM), Γ_1, that is constant for all Q-values. Γ_1 is related to the mean residence time between subsequent jumps, $\Gamma_1 \sim 1/\tau$, the proportionality depends on whether it is C_2 or C_3 rotation. Because of the normalization condition of the scattering function, it is $A_0(Q) = 1 - A_{10}$.

$A_0(Q)$ is the Q-dependent elastic incoherent structure factor (EISF) of the rotation which describes the spatial extension and the symmetry of the respective local motion. $A_0(Q)$ for rotations around the C_2 or C_3 symmetry axis is given for both cases by:

$$A_{0,c2} = A_{0,c3} = \frac{1}{2}\ [1 + j_0\left(Q\frac{2\sqrt{2}}{\sqrt{3}}d_{B-H}\right)]$$

d_{B-H} is the boron – hydrogen distance and $j_0(x)$ is the zero order Bessel function. Expressions for further rotational motions can be found in Ref. [4].

Vibrational motions are generally described by:

$$S_{vib}(Q,\Delta E) = D(Q)\delta(\Delta E) + [1 - D(q)S_{inel}(Q,\Delta E)]$$

where D(Q) denotes the Debye-Waller factor. In the harmonic and isotropic approximation, it is given by $D(Q) = \exp(\frac{<u^2>Q^2}{3})$, where $<u^2>$ is the mean square displacement of the hydrogen atom. In the one-phonon harmonic approximation, the inelastic scattering function $S_{inel}(Q,\Delta E)$ can be described by a damped harmonic oscillator.

From the measurements, it appears that vibrational and rotational motions are on similar energy scales. Therefore, for the analysis, the convolution of S_{rot} and S_{vib} had to be considered in full. The measured S(Q,ΔE) has thus been described using:

$$\begin{aligned} S_{meas}(Q,\Delta E) &= Res \otimes S_{rot} \otimes S_{vib} \\ &= Res \otimes [D(Q)A_0(Q)\delta(\Delta E) + D(Q)A_1(Q)L_1(\Gamma_1,\Delta E) + S_{inel}] \end{aligned} \quad \text{Eq. 2}$$

Res is the instrument resolution function measured at 3.5 K. The inelastic contribution has been modelled using a series of (over-) damped harmonic oscillators (DHO).

To focus on the low energy excitation spectrum, the data have also been converted to the imaginary part of the dynamical susceptibility, $-\chi''$ which is calculated (for system energy gain) from [5-7]:

$$\frac{1}{\pi}\chi'' = \frac{S(Q,-\Delta E)}{n_B}$$

n_B is the Bose occupation factor, $n_B = [\exp\left(\frac{\Delta E}{kT}\right) - 1]^{-1}$, and k is the Boltzmann constant. Data were summed over all Q-values.

For the data analysis of the measured QENS spectra, the dynamic susceptibility $-\chi''$ has been analysed to obtain the characteristic frequencies of the inelastic contributions.$-\chi''$ has been fitted by a weighted

sum of four damped harmonic oscillators (DHO) (plus background) whose imaginary part of the dynamical susceptibility have the form:

$$\chi''_{DHO} = \frac{E_D^2 \gamma_D \Delta E}{(E_D^2 - \Delta E^2)^2 + (\Delta E \gamma_D)^2}$$

E_D is the characteristic energy of the DHO and γ_D the damping factor. To account for the finite energy resolution of the instrument, the spectra was convoluted with a Gaussian resolution function of the appropriate width at the characteristic energy E_D obtained analytically from the instrument settings ([8]. The obtained values for E_D were taken as fixed input to reduce the number of free fit parameters in the analysis of *S(Q,ΔE).*

Appendix with Fig. A1 to A13

a)

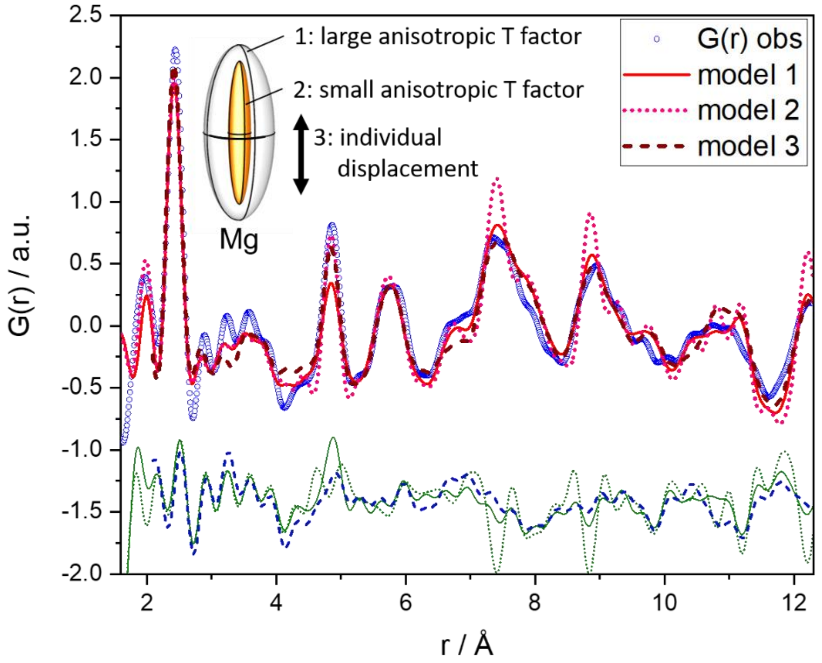

b)

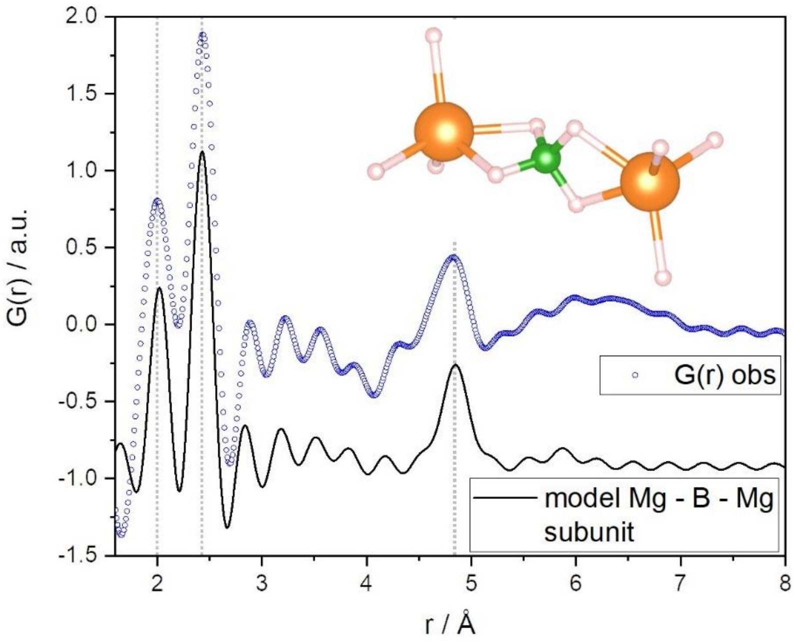

Figure A1 a) Comparison of calculated PDFs based on different models used to converge the local structure of γ-$Mg(BH_4)_2$. The inset shows a sketch of anisotropic temperature factors of Mg used in the corresponding models (50 % probability). b) The comparison modeled PDF of the "subunits" and the ball milled m sample.

SR-PXD of as-received γ-$Mg(BH_4)_2$

a) b)

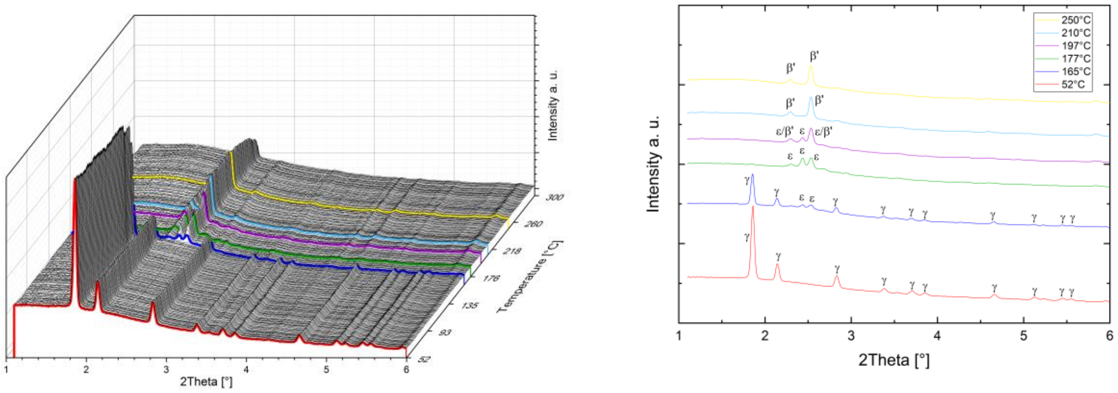

SR-PXD of ball milled $Mg(BH_4)_2$

c) d)

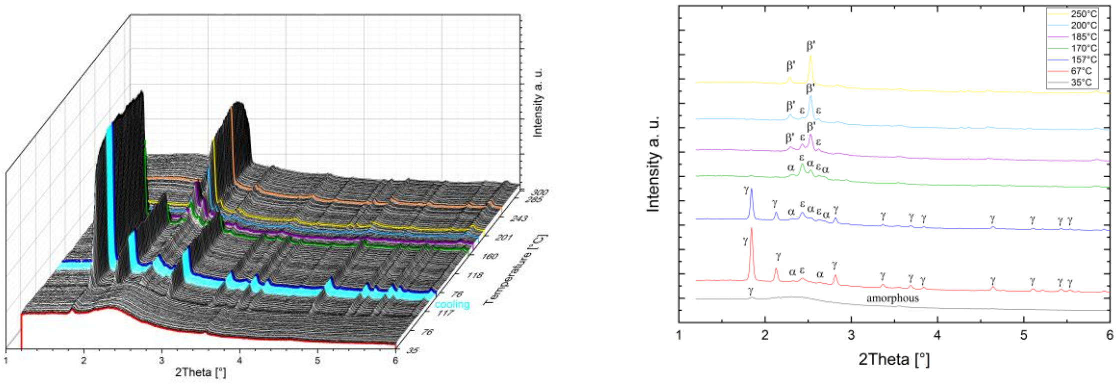

Figure A2 *in situ* SR-PXD data of $Mg(BH_4)_2$ samples while heating from 25 to 300 °C under 1 bar Ar. Coloured curves are a representation of different phases and are shown in these colours for better understanding in b) and d). a) and b) as-received γ-$Mg(BH_4)_2$, c) and d) ball milled $Mg(BH_4)_2$ with a cooling phase in between for the crystallization of γ-$Mg(BH_4)_2$, according to the experimental conditions used in this manuscript λ = 0.2254 Å.

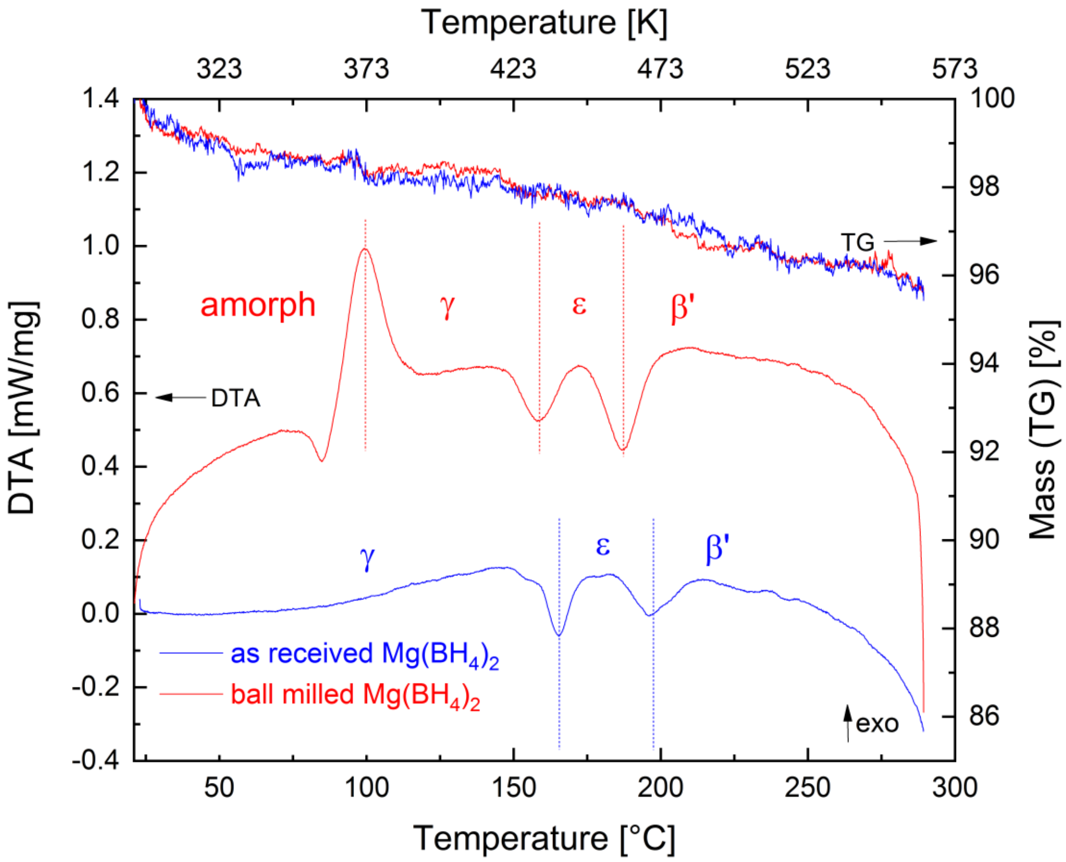

Figure A3 TG-DTA data of as-received and ball milled $Mg(BH_4)_2$ between RT and 285 °C heated at 5 °C min^{-1}. Dashed lines in DTA curves indicate peak temperatures for phase transitions of as-received γ-$Mg(BH_4)_2$ into ε-$Mg(BH_4)_2$ and β'-$Mg(BH_4)_2$ (black curve). Ball milled amorphous-$Mg(BH_4)_2$ transforms into γ-$Mg(BH_4)_2$ into ε-$Mg(BH_4)_2$ and β'-$Mg(BH_4)_2$ (yellow curve).

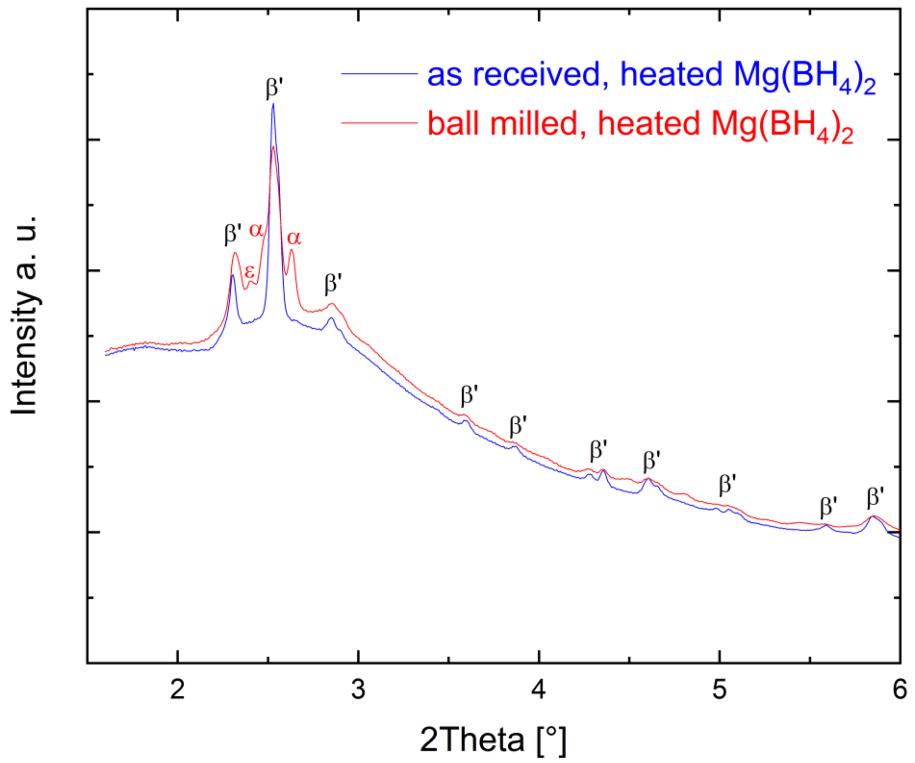

Figure A4 SR-PXD data at room temperature. a) as-received Mg(BH4)2 after heating to 550 K in beta' modification. B) ball milled Mg(BH4)2 after heating to 550 K in beta', alpha and epsilon modification.

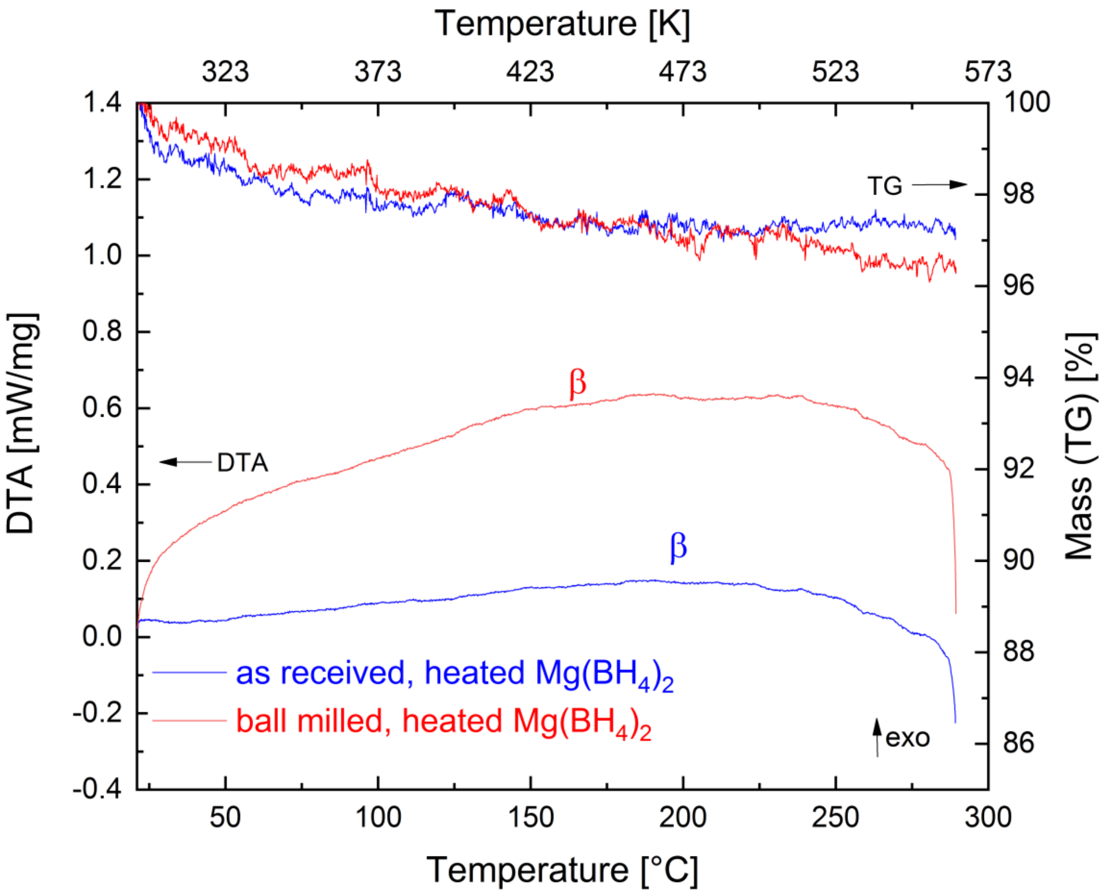

Figure A5 TG-DTA data of as-received and ball milled $Mg(BH_4)_2$ between RT and 285 °C heated at 5 °C min^{-1}. Dashed lines in DTA curves indicate peak temperatures for phase transitions of as-received γ-$Mg(BH_4)_2$ into ε-$Mg(BH_4)_2$ and β'-$Mg(BH_4)_2$ (black curve). Ball milled amorphous-$Mg(BH_4)_2$ transforms into γ-$Mg(BH_4)_2$ into ε-$Mg(BH_4)_2$ and β'-$Mg(BH_4)_2$ (yellow curve).

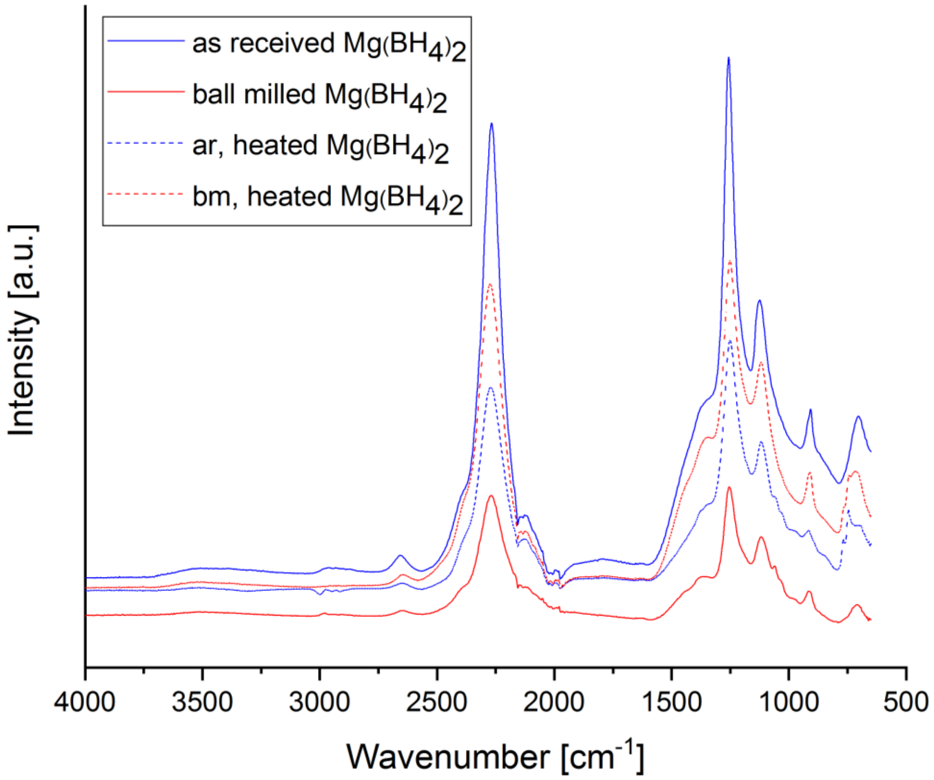

Figure A6 IR data at RT of as-received (ar), ball milled (bm) $Mg(BH_4)_2$ as well as samples which have been heated during QENS experiment to 550 K.

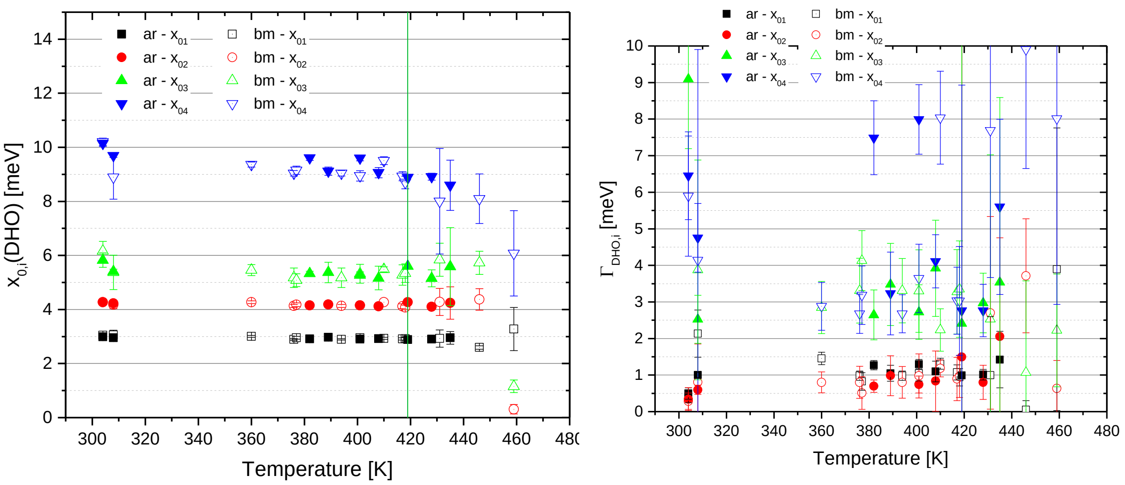

Figure A7 a) Peak position $x_{0,i}$ (i=1-4) of the damped harmonic oscillators (DHO) at measured temperatures. b) Half width half maximum of $\Gamma_{DHO,\ i}$ (i=1-4); solid symbols for as-received $Mg(BH_4)_2$, open symbols for ball milled $Mg(BH_4)_2$. As-received (ar) and ball milled (bm) $Mg(BH_4)_2$.

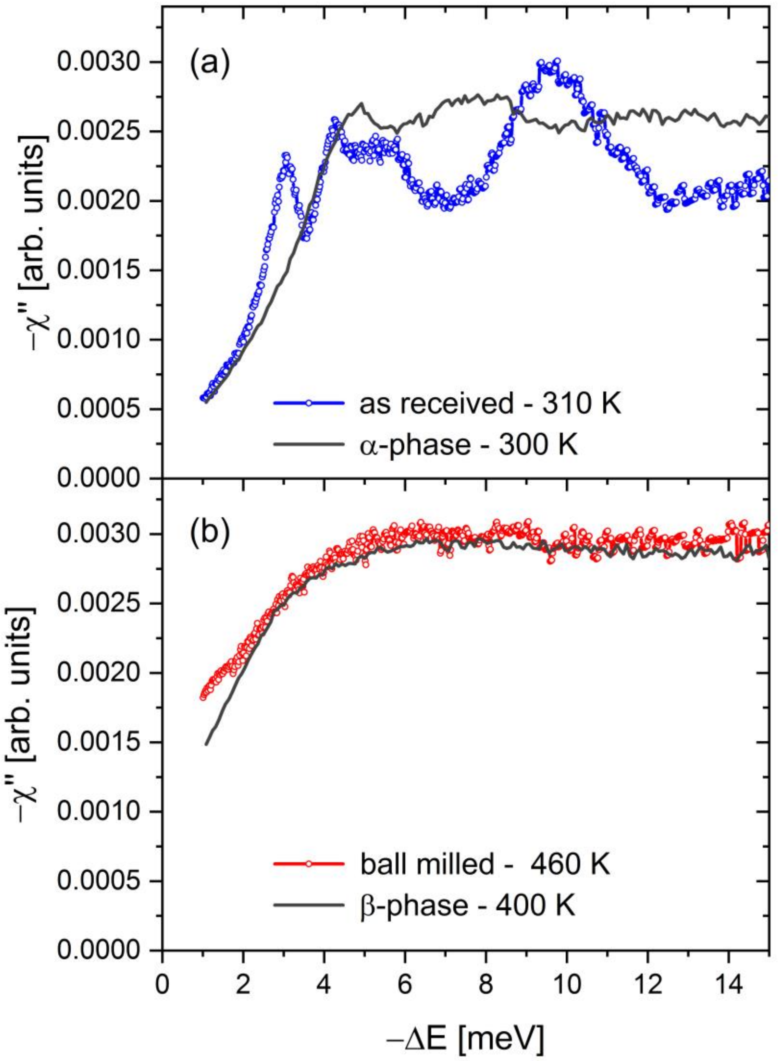

Figure A8 Dissipative part of the dynamical susceptibility –χ'' as a function of energy transfer –ΔE. a) as-received γ-$Mg(BH_4)_2$ compared to α-$Mg(BH_4)_2$ (from Ref. [1]) at 300 K); b) ball milled $Mg(BH_4)_2$ at 460 K shows no significant difference to β-$Mg(BH_4)_2$ (from Ref. [2] at 450 K). Blue circles: as-received γ-$Mg(BH_4)_2$. Red circles: ball milled $Mg(BH_4)_2$.

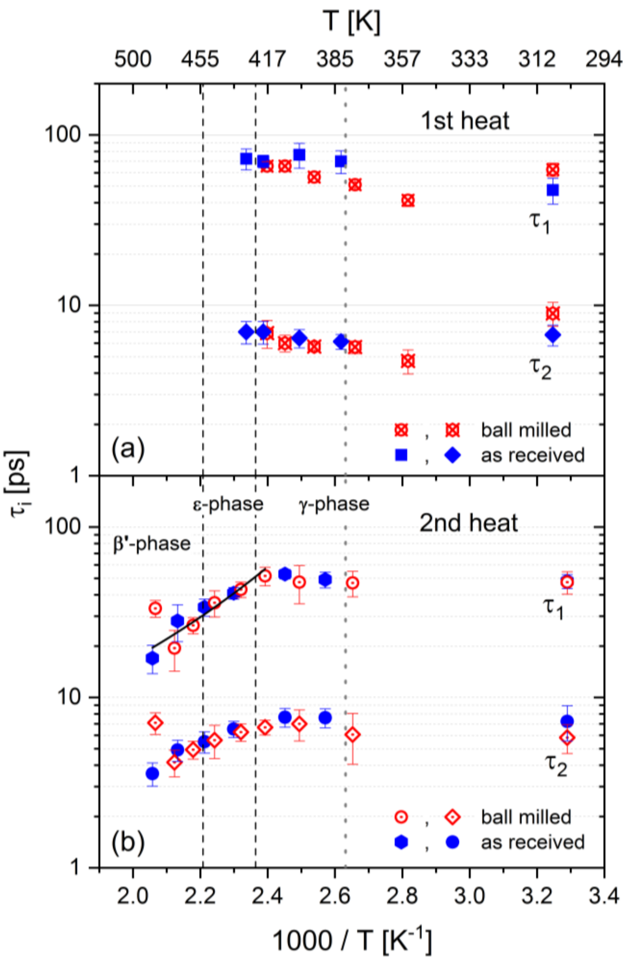

Figure A9 Characteristic timescales τ_i for the Lorentzians Γ_i (i = 1, 2). a) the temperature range was from 310 K but below the ε-phase transition, $T < T_{\varepsilon}$, b) temperature range was from 310 K to above the ε-phase transition, $T > T_{\varepsilon}$.

The fraction of hindered rotations p is plotted in **Fig. A10**. Heating of amorphous $Mg(BH_4)_2$ yields to a decrease of activated rotations up to the crystallization temperature. During the 2nd heat, the crystallized sample and γ-$Mg(BH_4)_2$ exhibit a continuous increase of activated BH_4 rotations until all C_2/C_3 modes are active (i.e. p = 0) at a temperature that coincides with the ε-phase transition temperature.

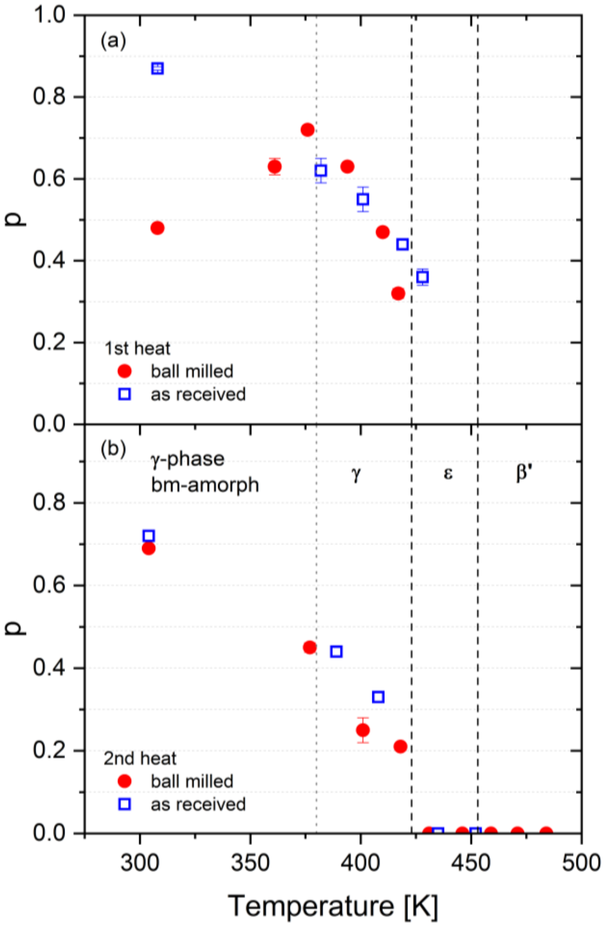

Figure A10 Fraction of the hindered rotations p derived from EISF and QISF. a) the temperature range was from 310 K but below the ε-phase transition, $T < T_ε$ b) temperature range was from 310 K to above the ε-phase transition, $T > T_ε$.

The mean square displacement (<u^2>) of the hydrogen atoms has been calculated from

$$I_{el} + I_{qe} = D(Q)(A_0 + A_1) = D(Q) = F\exp(\frac{< u^2 > Q^2}{3})$$

where F is the scaling factor. The results for <u^2> are shown in **Fig. A11**.

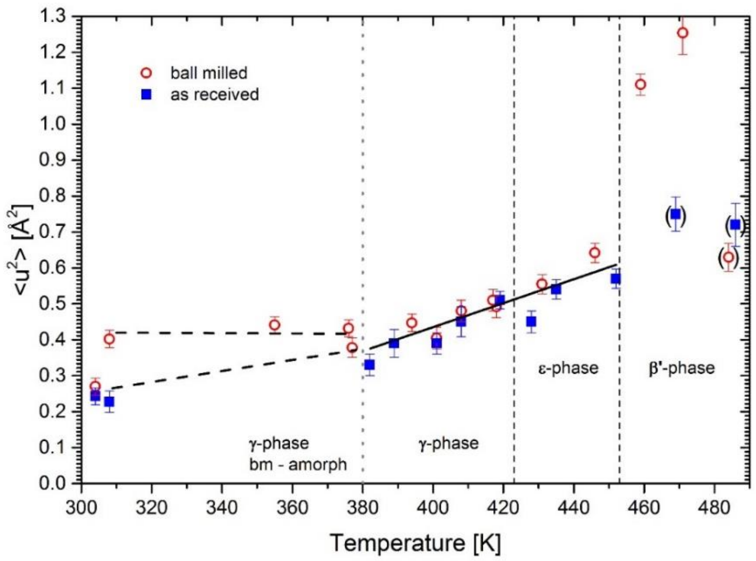

Figure A11 Mean square displacement <u^2> as a function of temperature for as-received γ-$Mg(BH_4)_2$ and ball milled (bm) $Mg(BH_4)_2$ between 300 and 490 K. Blue circles: as-received γ-$Mg(BH_4)_2$. Red squares: ball milled $Mg(BH_4)_2$. In brackets: as-received γ-$Mg(BH_4)_2$ lost hydrogen during heating.

In the beginning of the experiment, during the 1st heating, the difference between the two samples is obvious. The γ-$Mg(BH_4)_2$ shows an continuous increase in <u^2> while the <u^2> of the ball milled phase is larger, and remains almost constant upon heating to 380 K. In the amorphous material, fast vibrations appear to be enhanced compared to crystalline γ-$Mg(BH_4)_2$ while both samples appear to be quite similar in the temperature range 380 - 455 K. In the initially amorphous material, a jump like increase of <u^2> is observed at the phase transition to the β'. No such jump has been observed for γ-$Mg(BH_4)_2$, however, the latter showed signs of hydrogen loss during the QENS measurements at temperatures above 470 K and these data points will therefore not be discussed any further. The last data point of ball milled sample in the β'-modification at 485 K is also showing a decrease in <u_2>. However, the QENS data did not show a decrease in intensity, which would be indicative for hydrogen loss. SR-PXD data after heating, shown in Fig. A4, confirm the β'-$Mg(BH_4)_2$ phase at room temperature while TG data in Fig. A5 show a 2-3 wt.% hydrogen loss, which can explain the loss in intensity here [9].

The internal bending and stretching modes of the [BH_4] tetrahedra have been reported to be very similar for most of the polymorphs in $Mg(BH_4)_2$ and this trend is also shown in Fig. A6, with bending and stretching modes of the [BH_4] of four investigated samples (as-received, ball milled and two heated samples) are comparable, which can be explained by a very similar short range order.

The mean square displacement <u^2> reported for the solid state electrolyte $Mg(BH_4)_2$-diglyme$_{0.5}$ of the vibrational modes are showing a 'steplike increase' of $[BH_4]^-$ contributions at 320 K [10]. This was stated to result from the related phase transition of the sample. In our measurements, between RT and 450 K only linear dependencies were found for the three phase transitions (from amorphous into gamma, epsilon and beta'). A stepwise function was only found for the irreversible phase transition towards β'-phase. In agreement with literature, this behaviour was reported as well in α- to β-phase transition as well as in orthorhombic-$LiBH_4$ to hexagonal phase transition [1]. The latter is conductive [11]. Furthermore, a stepwise function was found in β-modification between 200 and 300 K [2]. Nevertheless, with those

results in mind, a correlation might be imaginable between the high temperature β and β'-phase and the reported phase transitions in $Mg(BH_4)_2$-diglyme$_{0.5}$.

Table S1 The dimensions and densities of the sample's pellets used for conductivity measurements. Densities taken from Ref. [12].

Sample	Weight (g)	Thickness (cm)	Diameter (cm)	Volume (cm^3)	Calculated Density (g/cm^3)	Theoretical Density (g/cm^3)	Relative Density (g/cm^3)
**γ-$Mg(BH_4)_2$**	0.150	1.21	15.5	0.228	0.648	0.54	1.19
**γ-$Mg(BH_4)_2$-BM**	0.112	1.34	12.5	0.164	0.681	0.54	1.25

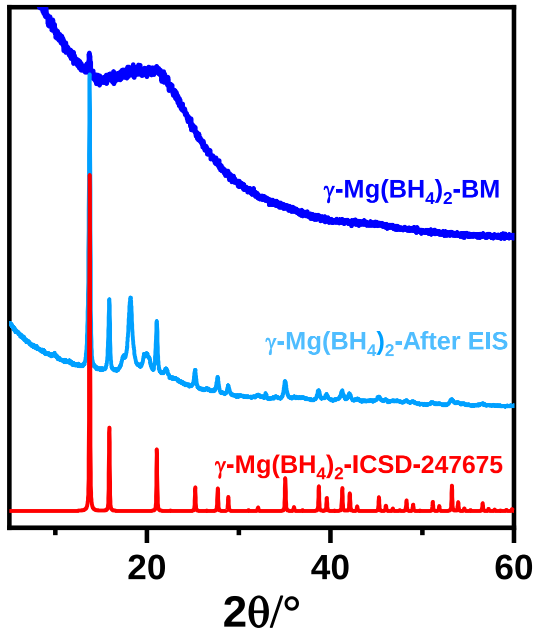

Figure A12 XPD of γ-$Mg(BH_4)_2$ after ball milling and EIS measurement and γ-$Mg(BH_4)_2$ from ICSD database with collection code 247675 (λ_{Cu} = 1.5406 Å). The additional peaks in the PXRD pattern of the γ-$Mg(BH_4)_2$ after EIS might be related to the formation of oxides. The pellet was exposed to air for 1 h after EIS measurement.

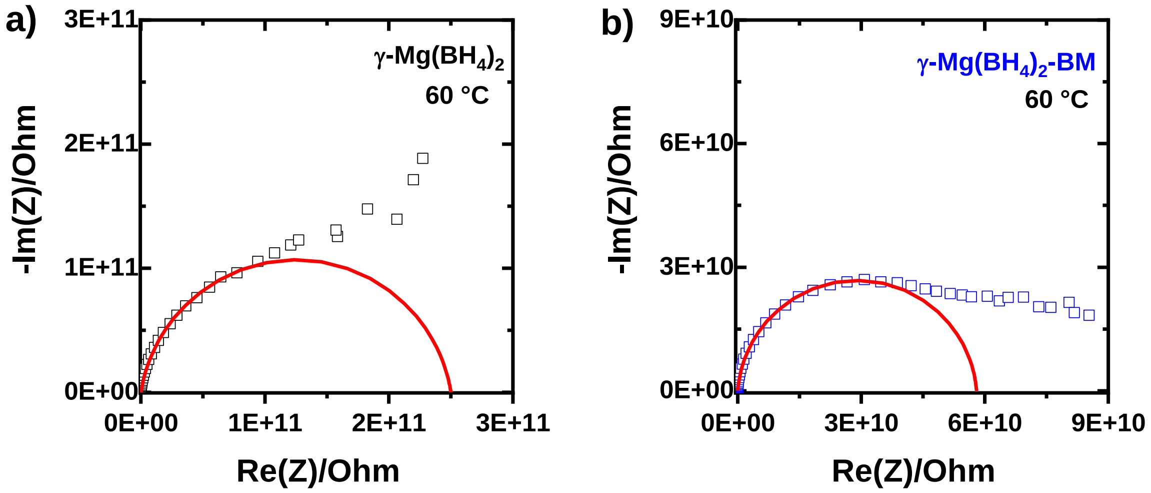

Figure A13 Nyquist plots of a) γ-$Mg(BH_4)_2$ and b) ball milled-$Mg(BH_4)_2$ at 60 °C. The squares represent the experimental data and the red lines are derived from the proposed electrical circuit model.

References

1 Silvi, L. *et al.* A quasielastic and inelastic neutron scattering study of the alkaline and alkaline-earth borohydrides LiBH4 and Mg(BH4)2 and the mixture LiBH4+ Mg(BH4)2. *Phys. Chem. Chem. Phys.* **21**, 718-728 (2019).

2 Silvi, L., Rohm, E., Fichtner, M., Petry, W. & Lohstroh, W. Hydrogen dynamics in [small beta]-Mg(BH4)2 on the picosecond timescale. *Phys. Chem. Chem. Phys.* **18**, 14323-14332, doi:10.1039/C6CP00995F (2016).

3 Martelli, P. *et al.* BH4− self-diffusion in liquid LiBH4. *The Journal of Physical Chemistry A* **114**, 10117-10121 (2010).

4 Hempelmann, R. *Quasielastic neutron scattering and solid state diffusion*. (Clarendon Press, 2000).

5 Arbe, A., De Molina, P. M., Alvarez, F., Frick, B. & Colmenero, J. Dielectric susceptibility of liquid water: Microscopic insights from coherent and incoherent neutron scattering. *Phys. Rev. Lett.* **117**, 185501 (2016).

6 Schober, H. An introduction to the theory of nuclear neutron scattering in condensed matter. *Journal of Neutron Research* **17**, 109-357, doi:10.3233/JNR-140016 (2014).

7 Kubo, R. The fluctuation-dissipation theorem. *Reports on progress in physics* **29**, 255, doi:https://doi.org/10.1088/0034-4885/29/1/306 (1966).

8 Unruh, T., Neuhaus, J. & Petry, W. The high-resolution time-of-flight spectrometer TOFTOF. *Nuclear Instruments and Methods in Physics Research Section A: Accelerators, Spectrometers, Detectors and Associated Equipment* **580**, 1414-1422 (2007).

9 Heere, M. *et al.* Effect of additives, ball milling and isotopic exchange in porous magnesium borohydride. *Rsc Advances* **8**, 27645-27653, doi:10.1039/c8ra05146a (2018).

10 Burankova, T. *et al.* Dynamics of the Coordination Complexes in a Solid-State Mg Electrolyte. *J Phys Chem Lett* **9**, 6450-6455, doi:10.1021/acs.jpclett.8b02965 (2018).

11 Maekawa, H. *et al.* Halide-stabilized LiBH4, a room-temperature lithium fast-ion conductor. *J Am Chem Soc* **131**, 894-895, doi:10.1021/ja807392k (2009).

12 Zavorotynska, O., El-Kharbachi, A., Deledda, S. & Hauback, B. C. Recent progress in magnesium borohydride Mg(BH4)(2): Fundamentals and applications for energy storage. *International Journal of Hydrogen Energy* **41**, 14387-14403, doi:10.1016/j.ijhydene.2016.02.015 (2016).
